# OP37 The Spanish Health Technology Assessment Network Methodological Approach For The Utilization Of Real-World Data In The Preadoption Phase

**DOI:** 10.1017/S0266462324000990

**Published:** 2025-01-07

**Authors:** Guillermo Pérez García, Celia Muñoz Fernandez, Lucía Prieto Remón, Carlos Tellería Orriols, Hugo Hernández Alemán, Soledad Isern De Val, Sandra García Armesto

## Abstract

**Introduction:**

Preadoption assessments are the most frequent type of evaluation conducted by the Spanish Network of Agencies for Health Technology Assessment and Services of the National Health System (RedETS). RedETS aimed to develop a framework to utilize real-world data (RWD) to better adjust its assessments to the Spanish population’s context and provide live assessments throughout the lifecycle of health technologies.

**Methods:**

A working group within RedETS was set up, which held several meetings to define the primary uses of RWD in the short term. Next, a manual review was conducted on national and international initiatives that provided guidance on the use of RWD in HTA. Common pathways for utilizing RWD in HTA were identified. The working group agreed to outline and explain the key overarching steps and provide general guidelines for working with RWD, developing as illustration a use case for an interventional technology. The Big Data project of Aragon (BIGAN) was chosen as the data source for the use case.

**Results:**

We formulated a case for leveraging RWD in the assessment of implantable cardiac defibrillators (ICDs) for the prevention of sudden cardiac death (SCD). Based on this scenario, we developed a methodological framework outlining a workflow consistent with RedETS practices. RWD complemented the usual process of systematic review of a technology. Crucial steps comprised the definition of data requirements through a data model specification, an exploratory data analysis, and the construction of a decision model. We presented solutions for dealing with unavailable data on essential variables and unstructured records. We discussed the main limitations to account for when working with RWD.

**Conclusions:**

The task ahead holds great hope but requires overcoming some challenges to fully deploy RWD-driven methods. This entails fostering collaboration with health authorities and designated data holders to address data access challenges. In the short term, it is essential to include data scientists in assessment teams and provide appropriate capacity-building to encompass RWD tools and modeling techniques.

